# Supplementary material for: Identification of photoperiod-induced specific miRNAs in the adrenal glands of Sunite sheep (Ovis aries)
Source: Front Vet Sci. 2022 Jul 22;9:888207. doi: 10.3389/fvets.2022.888207 (PMC9354845; doi:10.3389/fvets.2022.888207)
Supplement: Supplementary file 1 [file Data_Sheet_1.ZIP › Supplementary file/miRDeep2 file.zip!/miRDeep2 file/Novel_Structure/SP42A3/Novel_2.pdf]

Provisional ID	:	NC_019474.2_23943
Score total	:	1.2
Score for star read(s)	:	-1.3
Score for read counts	:	0
Score for mfe	:	0.9
Score for randfold	:	1.6
Score for cons. seed	:	
Total read count	:	30
Mature read count	:	30
Loop read count	:	0
Star read count	:	0

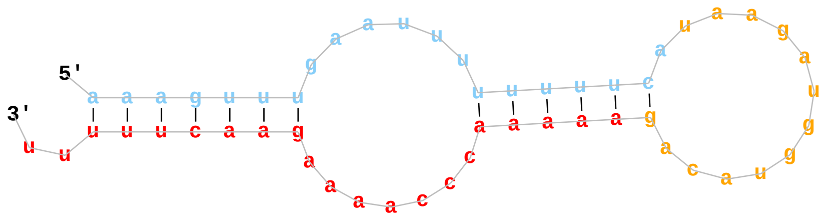

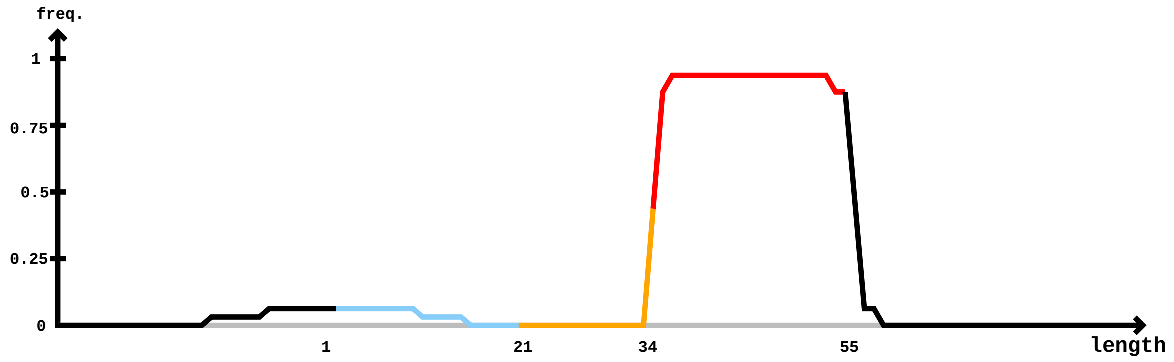

Star Mature

```
5'- gaguaucauaaauauuggguuggccaaaaaguuugaauuuuuuuucauaagaugguacagaaaaacccaaaagaacuuuuuggcuaaccuaguaccagcaguuugauucau   -3'    exp
    ((((........((((((((((((((((((((((......((((((.............)))))).......))))))))))))))))))))))...........))))..   reads      mm         sample
    ..............uuggguuggccaaaaaguuugC...........................................................................   1          1          seq
    ....................uggccaaaaaguuugaauuuu......................................................................   1          0          seq
    ............................................................aaaaacccaaaUgaacuuu................................   1          1          seq
    ............................................................aaaaacccaaaUgaacuuuuu..............................   13         1          seq
    .............................................................aaaacccaaaUgaacuuu................................   1          1          seq
    .............................................................aaaacccaaaUgaacuuuuu..............................   4          1          seq
    .............................................................aaaacccaaaUgaacuuuuug.............................   9          1          seq
    ..............................................................aaacccaaaUgaacuuuuuggc...........................   2          1          seq
```
